# Associations between Phthalate Exposure and Gestational Age at Delivery in a Diverse Pregnancy Cohort

**DOI:** 10.3390/toxics10120754

**Published:** 2022-12-03

**Authors:** Laura Sienas, Catherine Albright, Yu Ni, Adam Szpiro, Nicole R. Bush, Christine Loftus, Kurunthachalam Kannan, Frances Tylavsky, Catherine J. Karr, Kaja Z. LeWinn, Sheela Sathyanarayana

**Affiliations:** 1 Northwest Perinatal Associates, Portland, OR 97225, USA; 2 Department of Obstetrics and Gynecology, University of Washington, Seattle, WA 98195, USA; 3 Department of Epidemiology, University of Washington, Seattle, WA 98195, USA; 4 Department of Biostatistics, University of Washington, Seattle, WA 98195, USA; 5 Department of Psychiatry and Behavioral Sciences, University of California San Francisco, San Francisco, CA 94143, USA; 6 Department of Pediatrics, University of California San Francisco, San Francisco, CA 94143, USA; 7 Department of Environmental and Occupational Health Sciences, University of Washington, Seattle, WA 98195, USA; 8 Department of Environmental Health Sciences, New York University School of Medicine, New York, NY 10016, USA; 9 Department of Biostatistics and Epidemiology, University of Tennessee Health Science Center, Memphis, TN 38163, USA; 10 Seattle Children’s Research Institute, Seattle, WA 98101, USA; 11 Department of Pediatrics, University of Washington, Seattle, WA 98195, USA

**Keywords:** endocrine disrupting chemicals, environmental exposures, intimate partner violence, late preterm birth, phthalates, pregnancy, pregnancy stress, race

## Abstract

The association between prenatal phthalate exposure and late preterm birth (LPTB) is unclear. We examined singleton pregnancies (2006–2011) from a racially and socioeconomically diverse sample of women in the CANDLE cohort of the ECHO-PATHWAYS Consortium. Urine collected in the second and third trimester was analyzed for 14 phthalate metabolites. Multivariate logistic and linear regressions were performed for LPTB, defined as delivery 34–37 weeks, and gestational week, respectively. Models were controlled for socio-demographics, behavioral factors, clinical measurements, medical history, and phthalates in the other trimester. Effect modification by race and pregnancy stress, indicated by intimate partner violence (IPV), was investigated. We conducted a secondary analysis in women with spontaneous preterm labor. The rate of LPTB among 1408 women (61% Black, 32% White) was 6.7%. There was no evidence of decreased gestational age (GA) in association with any phthalate metabolite. Each two-fold increase in third trimester mono-benzyl phthalate (MBzP) was associated with 0.08 weeks longer gestational age (95% CI: 0.03, 0.12). When restricting to women with spontaneous labor, second trimester mono-n-butyl phthalate (MBP) was associated with 54% higher odds (95% CI: 2%, 132%) of LPTB. Associations were not modified by maternal race or IPV exposure. In conclusion, we observed mixed evidence concerning our hypothesis that prenatal phthalate exposure increases risk of LPTB, though secondary analyses suggest increased risk of spontaneous LPTB associated with MBP, which is consistent with a recent pooled analysis of 16 cohorts.

## 1. Introduction

Preterm birth (PTB), defined as delivery prior to 37 weeks’ gestation, is a common complication in pregnancy and a leading cause of neonatal morbidity [1]. Risk factors for spontaneous PTB include prior PTB, smoking, Black race, extremes of maternal age, genital tract infections, and low socioeconomic status [2]. The majority of preterm deliveries in the United States (US) occur in the late preterm period, defined as delivery greater than 34 weeks’ but less than 37 weeks’ gestation, with a rate of 7.1% in 2016 [3,4]. Infants of late preterm birth (LPTB) have increased morbidity compared to term infants given their developmental immaturity [5], but little is known about specific risk factors for LPTB [6,7]. Environmental factors, such as psychosocial stressors and chemical exposures, are associated with increased risk of PTB but have not been explored in specific relation to LPTB [1,8].

Phthalates are a class of chemicals found in 99–100% of pregnant women tested within the US National Health Nutrition and Examination Survey (NHANES) [9]. They are ubiquitous chemicals found in commonly used plastics and are contaminants in food production. Phthalates are also used as color stabilizers and fragrance in personal care products [10]. These chemicals are known to be endocrine disruptors and increase oxidative stress and inflammation [11]. Exposure has been associated with multiple adverse outcomes in adults and children such as increased reproductive toxicity in males, decreased thyroid function, and changes in pubertal development [12]. In a study including Black women in South Carolina, even after adjusting for socioeconomic status, they were found to have higher exposure to phthalates than other women. This may be due to higher concentrations of phthalates found in certain personal care products and other phthalate exposures [13,14].

Mechanistic pathways of preterm labor include pro-inflammatory cytokine release that leads to inflammation, increase in prostaglandins, or progesterone withdrawal [15]. Phthalates may play a role in initiating pro-inflammatory cytokines [16], activation of prostaglandin synthesis through peroxisome proliferator-activated receptors (PPAR) [17] and decreasing progesterone. This cascade theoretically could lead to preterm labor and delivery; however, the conclusions from prior studies evaluating prenatal phthalate exposure and preterm delivery are mixed [1,18,19,20,21,22,23,24]. Some studies have suggested an association with increasing maternal urinary phthalate concentrations and increased risk of preterm birth or oxidative stress [1,18,19,25], while other studies noted no association based on exposure assessed as maternal urinary phthalates [24], self-reported phthalate exposure [22] or amniotic fluid phthalate levels [23].

Maternal stress during pregnancy has also been associated with an increased risk of PTB [26,27,28]. Exposure to intimate partner violence, specifically, was associated with PTB in two systematic reviews [29,30]. Although the pathophysiology by which stress can increase risk is not completely defined, several pathways have been suggested such as increased TNF-α and IL-6 [26], increased oxidative stress [31,32], or increased corticotropin releasing hormone [33]. It is well known that maternal stress alters the hypothalamic-pituitary-adrenal axis, thus augmenting the risk of PTB [26,27,28]. Given greater stress and disparities in patients with lower socioeconomic status (SES) and non-white race, the physiological response to stress may be contributing to disparities in poor birth outcomes in the US [8].

The primary objective of this study is to evaluate the association between prenatal phthalate exposure and risk of LPTB as well as continuous gestational age at delivery. We evaluated if the association was modified by maternal race, as we hypothesized that given the higher burden of stressors Black women face, this may sensitize them to the adverse effects of phthalate exposures. We further explore whether associations are modified by prenatal stress, given the similar proposed mechanisms by which phthalates and stress may increase risk of PTB. We also perform a secondary analysis on women with spontaneous labor without medical indication for delivery to investigate the relationship of phthalate exposure on women with non-medically indicated births. We hypothesize that higher levels of phthalate concentration will be associated with greater risk for LPTB and that these associations will be stronger for Black women or for those exposed to IPV in pregnancy.

## 2. Materials and Methods

### 2.1. Study Participants

We included women enrolled in the Conditions Affecting Neurodevelopment and Learning in Early Childhood (CANDLE) study, described in depth previously [34,35]. In brief, 1503 pregnant women residing in Shelby County, Tennessee were recruited between 16 and 28 weeks’ gestation. Inclusion criteria were age 16–40 years old with a low medical-risk singleton pregnancy, English speaking, and intent to deliver at a participating hospital. Women provided informed consent before enrolling, and all research was approved by the University of Tennessee Health Science Institutional Review Board (IRB) as well as local institutional IRBs. This secondary analysis was approved by the University of Washington Human Subjects Division. In the current study, we included women-infant pairs in the CANDLE cohort with a live birth (six stillbirths excluded). Participants who did not have available data for gestational age at time of delivery (*N* = 47), or who had a baby with major congenital malformations (*N* = 12) were excluded. Because the pathophysiology leading to PTB less than 34 weeks is significantly different than that of LPTB [36], as well as the low incidence given recruitment of low medical risk singleton gestations, we also excluded births < 34 weeks (*N* = 30). The analytic sample for this study was 1408.

Demographic data, health questionnaires, stress surveys, and blood and urine samples were collected throughout pregnancy. Maternal race was obtained through self-report. In the third trimester, CANDLE assessed women’s exposure to IPV with four items from the Conflict Tactics Scale (CTS) [37], which asks whether the respondent experienced verbal, physical, or sexual abuse or injury from her partner in the past 12 months. Affirmative endorsements were summed for the total score (range = 0–4), representing the count of the types of IPV reported by the woman, and modeled continuously. The CTS has been shown to have good internal consistency and construct and discriminant validity [37]. The primary outcomes for this current study were continuous gestational age at birth and dichotomous LPTB (defined as delivery between 34 weeks 0 days and 36 weeks 6 days gestational age). Gestational age at enrollment was determined using the best obstetrical estimate, confirmed by patient report and medical record review. Birth outcomes were chart abstracted by study research nurses.

### 2.2. Phthalate Measurements

Urinary samples were collected twice from each woman during the second (mean: 23.0 weeks) and third trimester clinical visits (mean: 31.9 weeks). Specific gravity (SG) was measured with a handheld refractometer at the time of urine collection. Samples were collected in sterile, phthalate-free specimen cups, transferred to cryovials, and stored at −80 °C in the study repository (UTHSC Department of Pathology) until shipment for analysis. The phthalate metabolites present at detectable concentrations in at least 70% of the measurements at both time points were included in this analysis, including mono-isobutyl phthalate (MiBP), monoethyl phthalate (MEP), mono-methyl phthalate (MMP), mono-n-butyl phthalate (MBP), mono-benzyl phthalate (MBzP), mono-carboxy-isononyl phthalate (MCNP), mono-carboxy-isooctyl phthalate (MCOP), mono-(3-carboxypropyl) phthalate (MCPP), mono-(2-ethylhexyl) phthalate (MEHP), mono-(2-ethyl-5-hydroxyhexyl) phthalate (MEHHP), mono-(2-ethyl-5-oxohexyl) phthalate (MEOHP), mono-(2-ethyl-5-carboxypentyl) phthalate (MECPP), mono(4-hydroxypentyl) phthalate (MHPP) and mono(2-carboxymethylhexyl) phthalate (MCMHP). Analysis of phthalate metabolites involved enzymatic deconjugation, automated online solid phase extraction, separation with high performance liquid chromatography (HPLC), and detection by isotope-dilution tandem mass spectrometry. Process and instrument blanks were included for quality assurance. More details of the analytic method have been described elsewhere [38]. The limits of detection (LODs) were between 0.01 and 0.30 ng/mL. Measurements <LOD were replaced by LOD divided by the square root of 2 [39]. Metabolite concentrations were adjusted for specific gravity to account for urine dilution using the following Formula (1):

(1) $$P_{c}=P*\left[\frac{SG_{median}-1}{SG-1}\right]$$

where *P* is the measured urinary phthalate concentration, *SG* is the specific gravity for each participant, and $SG_{median}$ is the median *SG* over all samples. We further calculated the summed molar concentration across all DEHP metabolites (∑DEHP) as ∑DEHP = {[MEHP × (1/278.348)] + [MEHHP × (1/294.347)] + [MEOHP × (1/292.331)] + [MECPP × (1/308.330)] + [MCMHP × (1/308.33)]}. All the metabolites and ∑DEHP were natural log transformed, to achieve an approximately normal distribution, for analyses.

### 2.3. Statistical Analysis

Descriptive analyses of individual phthalate metabolites, gestational age, LPTB rate, and cohort characteristics were summarized as arithmetic means and standard deviations, counts and percentages, as appropriate.

In the primary analysis, multiple imputation by chained equations (MICE) was performed to address missingness of the individual phthalates (*N* = 288 in the second trimester [20%] and *N* = 339 in the third trimester [24%]) and covariates [40]. Each missing value was imputed 10 times with 100 iterations between each round of imputation. Associations between phthalates and odds of LTPB were estimated with logistic regression. Linear regressions with robust standard errors were used to estimate the associations between prenatal phthalates and gestational age. Separate models were used to analyze each individual phthalate metabolite and ∑DEHP in either visit. We decided a priori on several potential confounders to be included, and a hierarchical adjustment approach of four models was employed: Model 1 was adjusted for maternal race (Black vs. non-Black) and maternal age at delivery. Model 2 additionally controlled for maternal education (high school and less vs. above high school), income adjusted for household size [41], marital status (Married vs. Widowed/Divorced/Separated/Never married vs. Living with partner), insurance coverage (No insurance/Medicare or Medicaid Only vs. Medicare/Medicaid + Private/Private only), maternal SG-adjusted urinary cotinine levels in the second trimester, and medical history of preterm birth (Yes vs. No). Model 3 was considered our full model, and further adjusted for pre-pregnancy body mass index (BMI) class (underweight vs. normal vs. overweight vs. obese) and parity (0 vs. 1 or 2 vs. >=3). In addition, we mutually adjusted for individual phthalate in the other trimester in Model 4, to address potential confounding by exposure in other prenatal windows. For example, associations between second trimester MEP and outcomes were adjusted for covariates described above and third trimester MEP. We estimated effect modification by both maternal race and IPV using interaction models, by including cross-product terms in the full model.

In the secondary analysis, we restricted the study sample to those with a spontaneous labor (eight preterm deliveries with infant indications and 25 preterm deliveries with maternal indications were excluded) and repeated the analyses aforementioned. In the sensitivity analysis, we performed linear and logistic regressions with all adjustment models (Model 1–4) using complete data. Those without phthalate measurements in either visit were excluded. In addition, for any associations detected from Model 3 in the primary analysis, we evaluated evidence of nonlinear relationships in two ways: phthalate exposures were analyzed as quartiles instead of the continuous form, and generalized additive models (GAM) used to produce smooth nonlinear dose–response surfaces [42]. All analyses were conducted using STATA 15 (StataCorp, 2017, College Station, TX, USA) and RStudio 3.6.3 (R Cord Team, 2019, Vienna, Austria). A two-tailed *p*-value of ≤ 0.05 was considered statistically significant.

## 3. Results

The mean age of the 1408 women included in the current analysis was 26.5 years (SD: 5.4), and 61.0% self-identified as Black (Table 1).

At enrollment, 38.9% of the women had a household income less than $25,000, and 58.2% of them had a high school degree or less. Many of them (58.7%) were multiparous and the majority (92.1%) had no history of PTB. Two thirds of the women (66.7%) reported experiencing at least one type of abuse or injury by their partner over the past 12 months, with 48.8% of the overall population reporting one type of IPV and 1.8% reporting four unique types of IPV. We did not observe meaningful differences in baseline characteristics comparing the primary analytic sample and the sample with complete data (Table A1).

**Figure 1:**
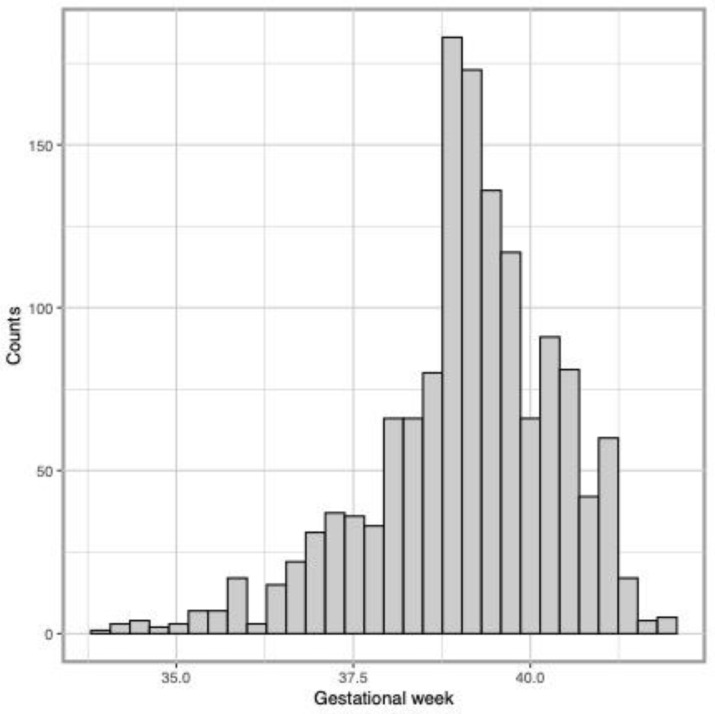
Distribution of gestational age at delivery in CANDLE participants.

The distribution of gestational week was slightly left-skewed with a mean of 39.1 (SD: 1.3) weeks (Figure 1), and the LPTB rate was 6.7%.

Phthalate metabolites included in the analysis were detected in 71.3–100% of samples (Table 2).

The Spearman correlations between phthalates in both visits ranged from 0.12 to 0.58. Majority (*N* = 13) of the 14 individual phthalates were higher in the second trimester than the third. MEP was found in highest concentration (geometric mean 133.2 ng/mL and 125.8 ng/mL in the second and third trimesters, respectively), whereas MHPP was the lowest (geometric mean 1.2 ng/mL and 0.4 ng/mL in the second and third trimesters, respectively). Phthalates were in similar concentrations to values found in the National Health and Nutrition Examination Survey (NHANES) 2009–2010 study [43], except for MEP which was found in higher concentration in our study samples. On average, Black women had higher concentrations of MEHP, MEP, MBzP, MBP and MiBP, while other metabolites were similar between Black and non-Black women (Table A2).

**Figure 2:**
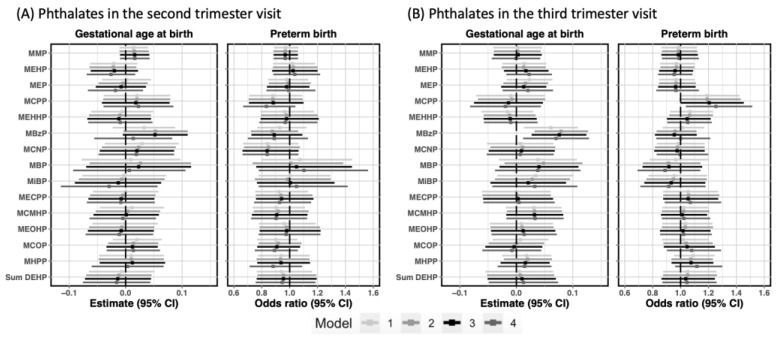
Estimated associations between prenatal phthalates in each visit and preterm birth in CANDLE participants. Model 1 adjusted for maternal race (Black vs. non-Black) and maternal age at delivery. Model 2 additionally controlled for maternal education (high school and less vs. above high school), income adjusted for household size, marital status (Married vs. Widowed/Divorced/Separated/Never married vs. Living with partner), insurance coverage (No insurance/Medicare or Medicaid Only vs. Medicare/Medicaid + Private/Private only), SG adjusted urinary cotinine measurements in the second trimester, and medical history of preterm birth (Yes vs. No). Model 3 was further adjusted for pre-pregnancy body mass index (BMI) class (Under vs. normal vs. overweight vs. obesity) and parity (0 vs. 1/2 vs. >=3). Model 4 was extensively adjusted for individual phthalates in the other trimester. The estimates were obtained from the models with continuous gestational age, and were interpreted as two-fold increase for each individual phthalate. The odds ratio was interpreted as two-fold increase for each individual phthalate.

We did not observe significant inverse relationships between individual phthalates in each visit and gestational age at birth (Figure 2).

**Figure A1:**
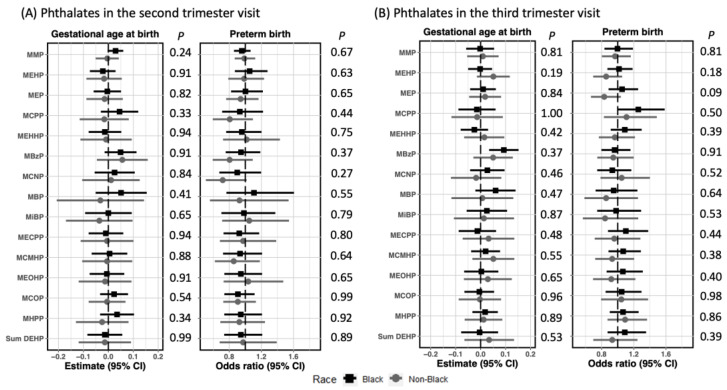
Estimated associations between prenatal phthalates and birth outcomes from the fully adjusted models, stratified by maternal race. The interaction models were adjusted for maternal age at delivery, maternal education (high school and less vs. above high school), income adjusted for household size, marital status (Married vs. Widowed/Divorced/Separated/Never married vs. Living with partner), insurance coverage (No insurance/Medicare or Medicaid Only vs. Medicare/Medicaid + Private/Private only), SG adjusted urinary cotinine measurements in the second trimester, medical history of preterm birth (Yes vs. No), pre-pregnancy body mass index (BMI) class (Under vs. normal vs. overweight vs. obesity), parity (0 vs. 1/2 vs. >=3), and the interaction term between maternal race (Black vs. non-Black) and individual phthalates. The estimates were obtained from the models with continuous gestational age, and were interpreted as two-fold increase for each individual phthalate. The odds ratio was interpreted as two-fold increase for each individual phthalate. The *p* value is for the interaction term.

**Figure A2:**
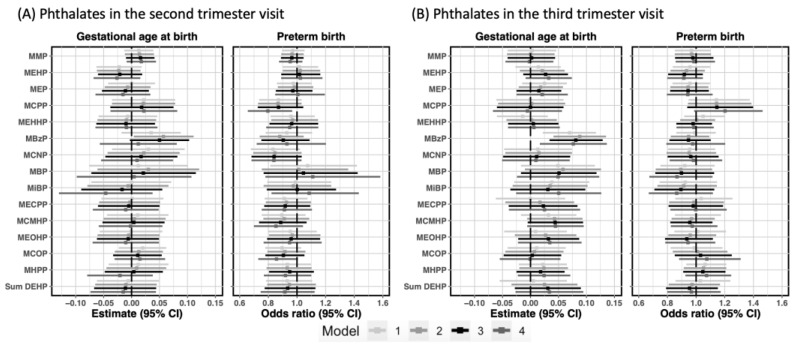
Estimated associations between prenatal phthalates in each visit and birth outcomes in CANDLE participants with complete data of phthalate measurements in either visit and birth outcome. Model 1 adjusted for maternal race (Black vs. non-Black) and maternal age at delivery. Model 2 additionally controlled for maternal education (high school and less vs. above high school), income adjusted for household size, marital status (Married vs. Widowed/Divorced/Separated/Never married vs. Living with partner), insurance coverage (No insurance/Medicare or Medicaid Only vs. Medicare/Medicaid + Private/Private only), SG adjusted urinary cotinine measurements in the second trimester, and medical history of preterm birth (Yes vs. No). Model 3 was further adjusted for pre-pregnancy body mass index (BMI) class (Under vs. normal vs. overweight vs. obesity) and parity (0 vs. 1/2 vs. >=3). Model 4 was extensively adjusted for individual phthalates in the other trimester. The estimates were obtained from the models with continuous gestational age, and were interpreted as two-fold increase for each individual phthalate. The odds ratio was interpreted as two-fold increase for each individual phthalate.

**Figure A3:**
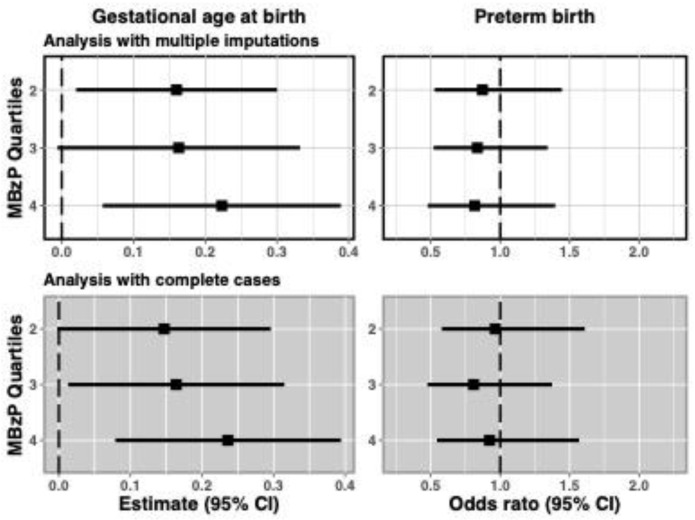
Estimated association between MBzP quartiles in the 2nd visit and birth outcome in overall CANDLE participants and participants with complete data of phthalate measurements in either visit and birth outcome. The models were adjusted for maternal race (Black vs. non-Black), maternal age at delivery, maternal education (high school and less vs. above high school), income adjusted for household size, marital status (Married vs. Widowed/Divorced/Separated/Never married vs. Living with partner), insurance coverage (No insurance/Medicare or Medicaid Only vs. Medicare/Medicaid + Private/Private only), SG adjusted urinary cotinine measurements in the second trimester, medical history of preterm birth (Yes vs. No), pre-pregnancy body mass index (BMI) class (Under vs. normal vs. overweight vs. obesity), and parity (0 vs. 1/2 vs. >=3). The estimates were obtained from the models with continuous gestational age, and were interpreted as two-fold increase for each individual phthalate. The odds ratio was interpreted as two-fold increase for each individual phthalate.

**Figure A4:**
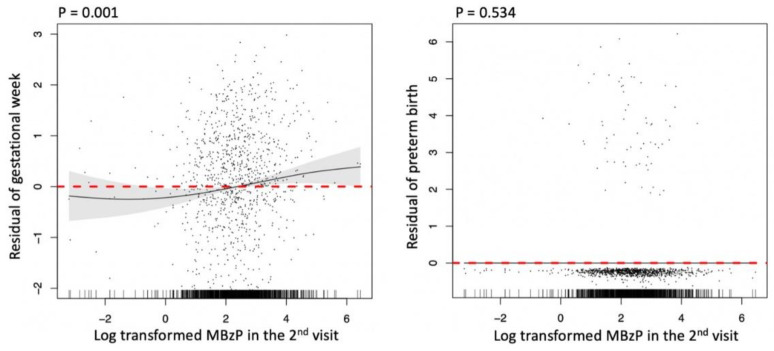
Estimated association between MBzP in the 2nd visit and birth outcome in overall CANDLE participants with complete data of phthalate measurements in either visit and birth outcome from the generalized additive models. The models were adjusted for maternal race (Black vs. non-Black), maternal age at delivery, maternal education (high school and less vs. above high school), income adjusted for household size, marital status (Married vs. Widowed/Divorced/Separated/Never married vs. Living with partner), insurance coverage (No insurance/Medicare or Medicaid Only vs. Medicare/Medicaid + Private/Private only), SG adjusted urinary cotinine measurements in the second trimester, medical history of preterm birth (Yes vs. No), pre-pregnancy body mass index (BMI) class (Under vs. normal vs. overweight vs. obesity), and parity (0 vs. 1/2 vs. >=3).

For each 2-fold increase in the second trimester MBzP concentrations, we observed a positive association of 0.05 more gestational weeks (β: 0.05, 95% CI: −0.01, 0.11) while each 2-fold increase in the third trimester was associated with 0.08 more gestational weeks (β: 0.08, 95% CI: 0.03, 0.12). In the model with mutual adjustment for both trimesters, the association remained only for the third trimester (β: 0.07, 95% CI: 0.01, 0.13). When LPTB was analyzed as a dichotomous outcome, we observed increased odds of LPTB (OR: 1.21, 95% CI: 1.00, 1.45) in relation to MCPP in the third trimester, which was strengthened when adjusting for MCPP in the second trimester (OR: 1.25, 95% CI: 1.04, 1.51). Nevertheless, in the mutually adjusted model, the association between second trimester MCPP and LPTB reversed to negative. Evaluation of associations between phthalates in each visit and birth outcomes were not modified by maternal race (Figure A1). No modification by IPV for the associations of interest was found (Table A3). When the analyses of individual phthalates and GA/LPTB were repeated using complete data, findings agreed with those obtained using multiple imputation (Figure A2). When MBzP in the third trimester was divided into quartiles, we found longer gestation in women with MBzP in the 2nd to 4th quartiles compared to women with MBzP in the 1st quartile (Figure A3). The smooth effect curves generated from GAMs based on complete data (Figure A4) was consistent with a linear trend for the association between MBzP in the third trimester and gestational week (*p*: 0.001).

**Figure 3:**
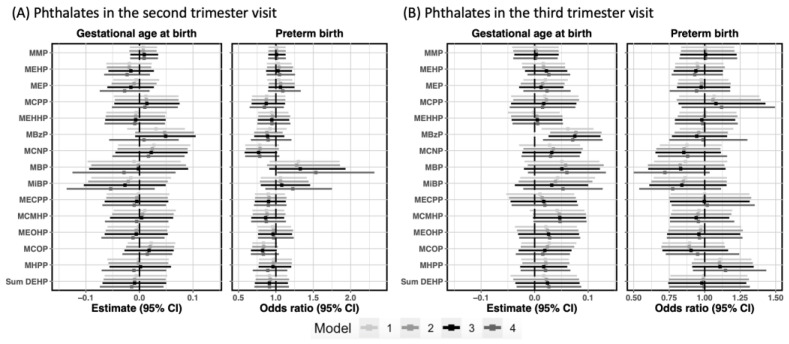
Estimated associations between prenatal phthalates in each visit and birth outcomes in CANDLE participants with a spontaneous labor. Model 1 adjusted for maternal race (Black vs. non-Black) and maternal age at delivery. Model 2 additionally controlled for maternal education (high school and less vs. above high school), income adjusted for household size, marital status (Married vs. Widowed/Divorced/Separated/Never married vs. Living with partner), insurance coverage (No insurance/Medicare or Medicaid Only vs. Medicare/Medicaid + Private/Private only), SG adjusted urinary cotinine measurements in the second trimester, and medical history of preterm birth (Yes vs. No). Model 3 was further adjusted for pre-pregnancy body mass index (BMI) class (Under vs. normal vs. overweight vs. obesity) and parity (0 vs. 1/2 vs. >=3). Model 4 was extensively adjusted for individual phthalates in the other trimester. The estimates were obtained from the models with continuous gestational age, and were interpreted as two-fold increase for each individual phthalate. The odds ratio was interpreted as two-fold increase for each individual phthalate.

When restricting the study population to those with a spontaneous labor, we observed similar associations with MBzP as those in the primary analysis. An association between MBzP in the third trimester and longer gestation was detected in the overall sample (β: 0.08, 95% CI: 0.03, 0.12) (Figure 3).

One unique finding in this restricted population was an increased odds of LPTB (OR: 1.54, 95% CI: 1.02, 2.32) per two-fold increase in second trimester MBP, after third trimester MBP was controlled. No other associations were observed. As in the primary analysis, we did not observe evidence of modification by IPV or maternal race (Table A4).

## 4. Discussion

In a large diverse study population that is underrepresented in the literature, we observed mixed results in the association between prenatal phthalate exposure and gestational age at time of delivery but some similar results to a recently published 16 study pooled analysis examining prenatal phthalate exposure and preterm births [25] in which higher prenatal MCPP and MBP exposure was significantly associated with an increased risk of preterm birth. We observed no adverse relationships between phthalate exposure and continuous GA at delivery in the combined spontaneous and medically indicated births cohort after robust control for covariates. We observed a small positive relationship between third trimester MBzP and gestational age at delivery (0.08 more gestational weeks for every 2 fold increase in MBzP). There is also an association between third trimester MCPP and increased risk of LPTB (OR: 1.21, 95% CI: 1.00, 1.45), which was strengthened when adjusting for the second trimester MCPP concentration. In restricting to spontaneous labor, exposure to MBP increased odds of spontaneous LPTB by 54% for every 2-fold increase of MBP. There was no evidence that associations varied by maternal race or IPV.

The primary findings of our study only partially agree from four previous studies that suggest that prenatal phthalate exposure is linked to a reduction in gestational age and increased risk of preterm birth [1,18,19,25,44]. Our analysis of spontaneous LPTB does agree with the associated increased risk of PTB with exposure to MBP in two unique cohort studies and the recent 16 cohort pooled analysis [1,18,25,44]. Additionally, we observed an association of MCPP (while adjusting for second trimester exposure) with increased rates of LPTB. This has been noted previously in a restricted sample of spontaneous preterm birth patients and now the pooled study [1,25]. Reasons for discrepancies in other findings may be related to associations with LPTB vs. overall preterm birth and our larger sample size and robust control for covariates not normally available in most epidemiologic analyses. In addition, the study population is more diverse in educational status, income, race and martial status than most of those included in previous reports. In two studies [1,18], Ferguson et al. found MEHP, MECPP, and ∑DEHP metabolites were associated with increased odds of preterm birth and MBzP, MBP, MiBP with increased markers of oxidative stress When particularly evaluating the women with spontaneous PTB (*N* = 57), Ferguson noted the association with MBP and decreased gestational age [1]. This study cohort was smaller (*N* = 482) and comprised of mostly White and privately insured patients. A pooled study of 16 prospective cohorts, totaling 6000 pregnant patients, found that MBP, MiBP, MECPP, MCPP were associated with increased odds of PTB (12–16%) [25]. This is the largest cohort study to date, but the authors were unable to separate between spontaneous and indicated preterm birth. Additionally, this cohort was not restricted to LPTB. In a study conducted in Mexico City (*N* = 60), Meeker et al. [19] observed that MBP, MBzP and 5 metabolites of DEHP increased risk of preterm delivery. This cohort was predominantly LatinX included both spontaneous and medically indicated preterm birth, and urine samples were taken at a later gestational age. A cohort in Puerto Rico, DBP and DiBP metabolites were associated with increased spontaneous preterm birth. This included MBP, which was associated with 1.55 days shorter gestation [44]. Our study suggested an adverse risk of MBP only in women with spontaneous LPTB and did not find this association in the overall cohort.

Others have reported no associations between maternal phthalate exposures and preterm birth [22,23,24]. In a low income and underrepresented minority population in Rotterdam, The Netherlands (*N* = 6302), Burdorf [22] did not find significant associations however exposure was determined based on self-reported occupational exposures, which are less accurate than biomarker analyses. Huang et al. [23] found no correlation between amniotic fluid phthalate levels and preterm birth in a study of 65 women in Taiwan. Lastly, in a design using maternal urinary phthalates for exposure characterization as done in our study, Suzuki et al. [24] analyzed maternal urinary phthalates and preterm birth in Japan in a cohort of 149 women, and also observed no associations. Notably, this study had a small sample size and low preterm birth rate in the population (1.5%).

In the current literature, only one study [45] examined the joint impact of phthalates and maternal stress on preterm birth, finding that increased maternal stress modified the association with PTB by increasing effect estimates. No studies have examined stress-phthalate interactions in the prediction of LPTB. Contrary to our hypothesis, we did not observe evidence of modification by maternal stress in this study population. We also explored effect modification by maternal race. Although small differences by maternal race were observed in the relationships between phthalates and preterm birth outcomes, effect modification was not evident. While not statistically significant, the minor differences observed may be due to social factors such as racism and other socioeconomic stressors that could affect susceptibility. This analysis was not designed to be able to further disentangle these complex relationships.

Our results in the CANDLE population were largely null for the combined spontaneous and medically indicated preterm birth subsample, but findings of MBP related to higher odds of preterm birth in spontaneous births only were consistent with four other cohort studies [1,18,19,44]. We observed a statistically significant but modest increase in gestational length associated with third trimester MBzP in both the full cohort and women with spontaneous LPTB. When considering the unexpected finding with MBzP, we identified some literature demonstrating MBzP can activate PPAR-gamma [17], which is a receptor that has anti-inflammatory properties in the preterm birth cascade pathway [46]. Further study is needed on the affinity of activation by MBzP and the impact of PPAR-gamma on altering the LTPB pathway. Two previous studies [20,21] have also noted a positive relationships between prenatal phthalate exposure and gestational age at time of delivery such as this study noted with MBzP. Adibi et al. [20] noted that 75th percentile of ∑DEHP had a prolongation association of 2 days gestation in their study compared to the 25th percentile. Given that DEHP is the aggregate measure of several metabolites, this study could not identify which of the three studied metabolites was driving this association. Wolff et al. [21] found a protective association of all low molecular weight metabolites on gestational age at delivery. Of note, the study population was over 50% of women being Hispanic ethnicity.

Exposure to phthalates is ubiquitous in our daily environment [24]. The risk of spontaneous LPTB is increased with exposure to MBP. Higher levels of MBP have been detected in pregnant women that used eye makeup, sunscreen, nail polish and hair nutrient products [47]. By limiting use of these products in pregnancy, women may decrease their exposure to MBP and risk of spontaneous LPTB. Exposure to maternal stress through IPV in pregnancy does not appear to impact the relationship between phthalates and LPTB in this population.

Previous studies have shown that modification and reduction of exposure to all endocrine disruptors may be beneficial in pregnancy [10,25,48]. Using G computation in the pooled analysis, hypothetical reductions in phthalate levels were associated with decreased rates of PTB that were dose-dependent [25]. The best evidence of link to preterm birth is with more thoroughly studied endocrine disrupting chemicals such as lead [49,50]. and mercury [51]. Although this study showed mixed results of the association of phthalates on LPTB, overall reduction in phthalate exposure can help decrease other more well established adverse maternal and neonatal health outcomes [52,53,54,55].

This study highlights the importance of expanding the literature to include more ethnically diverse, well characterized and prospective cohort studies. Our cohort, with a large prevalence of risk factors for PTB, such as non-White race and low SES, highlights that there may be other factors that contribute much more to the attributable risk of LTPB in such a diverse patient population. Finally, to address the possibility of critical windows of phthalate exposure increasing the risk of LPTB, our study incorporated multiple measures of urinary phthalates across pregnancy, allowing examination of variation in exposure and excretion that may occur across pregnancy. If a critical window for phthalate exposure exists, future studies could then evaluate risk of LPTB as well as early term birth, a time period which also causes increased neonatal morbidity.

This is the most diverse prospective cohort study in the current literature on phthalate exposure and preterm delivery. The CANDLE cohort includes a large proportion of Black women in the urban Southeastern US, an underrepresented population in the literature. We were able to collect urine samples from two time points to assess the congregate risk of phthalate exposure over pregnancy. Another strength is that we adjusted for a comprehensive set of control variables, including many risk factors for preterm delivery, reducing the likelihood that observed associations were impacted by residual confounding. We were additionally able to perform a secondary analysis on women with spontaneous labor, as this may have different mechanistic implications to phthalate exposure.

Limitations of this study include there was minimal maternal medical comorbidity data collected for these pregnancies, which may impact the pathophysiology of labor. Given CANDLE enrollment of low-risk pregnancies, fewer women delivered preterm. This reduced variability in the outcome may have reduced our chances of detecting a true association. Because phthalates were measured only for mother-child dyads who attended clinic visits in early childhood, exposure was missing for several women. We addressed this using multiple imputation and compared all findings to those obtained using complete case analysis; in all cases we obtained similar results. Finally, it is possible that phthalate exposure does increase risk of LPTB, but our study failed to detect this association. Exposure to phthalates may need to occur at a critical time window in pregnancy to increase risk for LPTB. Given that urinary phthalate assessment occurred only twice per pregnancy at a non-standardized gestational age, we may have been unable to detect this exposure window. Alternatively, a spot urine may not represent integrated exposures during pregnancy, which may increase risk of LPTB.

## 5. Conclusions

We observed mixed evidence concerning our hypothesis that environmental phthalate exposure in this population at high risk for preterm birth. Exposure does not appear to increase risk for LPTB for all births combined, but exposure to the MBP metabolite was associated with an increased risk of spontaneous LPTB, which is consistent with multiple cohort studies. We observed no modification by maternal race or exposure to IPV in this association. Further research should continue to explore additional environmental exposures in all populations to find potential meaningful behavioral and policy changes that could decrease exposures and improve health outcomes.

## Figures and Tables

**Table 1:** Baseline characteristics of CANDLE participants (*N* = 1408).

Variables	Total (*N* = 1408)	
	N/Mean	%/SD
Maternal age (years)	26.4	5.4
Race		
African American	859	61.0%
White	454	32.2%
Asian	13	0.9%
American Indian/Alaska Native	1	0.1%
Multiple	6	0.4%
Other	73	5.2%
Missing	2	0.1%
Education level		
<High School	173	12.3%
High School/GED	646	45.9%
Technical School	132	9.4%
College Degree	288	20.5%
Grad/Professional Degree	167	11.9%
missing	2	0.1%
Marital status		
Married	544	38.6%
Widowed/Divorced/Separated/Never married	603	42.8%
Living with partner	260	18.5%
missing	1	0.1%
Household income		
$0–$24,999	548	38.9%
$25,000–$44,999	248	17.6%
$45,000–$74,999	267	19.0%
>=$75,000	222	15.8%
missing	123	8.7%
Health insurance coverage		
No insurance	2	0.1%
Medicaid and/or Medicare only	793	56.3%
Medicaid/Medicare and Private	39	2.8%
Private Only	574	40.8%
Parity		
0	582	41.3%
1–2	648	46.0%
3–5	168	11.9%
>=6	10	0.7%
Prior preterm birth(s)		
0	1297	92.1%
1	96	6.8%
2	11	0.8%
3	4	0.3%
Pregnancy urinary cotinine (ng/mL)	64.1	316.7
Pre-pregnancy BMI (kg/m^2^)	27.5	7.5
Intimate partner violence (count of types of violence reported)		
0	388	27.6%
1	687	48.8%
2	154	10.9%
3	73	5.2%
4	25	1.8%
Missing	81	5.8%

Abbreviations: BMI, Body Mass Index; GED, Graduate Equivalency Degree.

**Table 2:** Distributions of specific gravity adjusted urinary phthalate concentrations in CANDLE.

Metabolites	Percent Detected	Minimum	25th Percentile (ng/mL)	Median (ng/mL)	75th Percentile (ng/mL)	Maximum	Geometric Mean (ng/mL) (SD)
The second trimester visit							
MMP	72.32%	0.06	0.3	5.64	13.21	201.78	2.47 (7.81)
MEHP	99.11%	0.01	4.36	7.47	13.71	627.91	7.66 (3.36)
MEP	99.91%	0.06	57.44	129.26	307.41	11710.34	133.15 (3.39)
MCPP	99.82%	0.06	1.37	2.05	3.5	111.23	2.38 (2.33)
MEHHP	99.91%	0.01	18.21	27.75	47.47	2225.81	31.85 (2.44)
MBzP	99.73%	0.03	12.16	20.23	38.38	1013.05	21.58 (2.56)
MCNP	99.91%	0.01	2.1	3.28	5.46	113.44	3.58 (2.2)
MBP	100.00%	0.39	22.03	31.59	48.23	713.09	32.48 (1.92)
MiBP	99.91%	0.07	8.21	12.66	19.05	495.58	13.15 (2.04)
MECPP	99.91%	0.01	12.07	18.14	31.06	1607.01	20.72 (2.36)
MCMHP	99.91%	0.06	11	17.33	31.37	695.49	19.76 (2.4)
MEOHP	99.91%	0.03	8.85	13.34	23.42	1050.13	15.57 (2.39)
MCOP	99.91%	0.02	6.7	12.66	31.54	2405.67	15.93 (3.31)
MHPP	98.21%	0.04	0.66	1.18	2.11	33.67	1.22 (2.42)
Sum DEHP	NA	0.00	0.2	0.29	0.51	19.77	0.34 (2.36)
The third trimester visit							
MMP	89.24%	0.04	1.4	2.38	3.91	190.86	2.02 (3.51)
MEHP	80.36%	0.03	0.73	2.28	5.29	417.27	2.16 (4.15)
MEP	100.00%	3.37	46.69	110.6	309.57	22148.62	125.82 (3.86)
MCPP	99.91%	0.03	0.9	1.41	2.32	85.14	1.52 (2.35)
MEHHP	99.25%	0.01	5.04	8.16	14.23	724.46	8.67 (3)
MBzP	98.60%	0.04	5.49	10.33	19.47	634.34	10.14 (3.05)
MCNP	99.63%	0.01	0.28	0.47	0.87	32.30	0.52 (2.5)
MBP	100.00%	1.70	9.54	15.94	25.64	490.24	15.98 (2.12)
MiBP	100.00%	0.34	4.35	7.73	12.39	137.00	7.55 (2.19)
MECPP	100.00%	0.79	7.59	11.79	19.44	538.77	13.2 (2.41)
MCMHP	98.69%	0.02	3.88	6.15	10.96	302.42	6.71 (2.89)
MEOHP	99.91%	0.05	3.76	6.2	10.2	472.08	6.82 (2.56)
MCOP	99.81%	0.05	1.15	2.18	5.04	147.33	2.52 (3.04)
MHPP	71.28%	0.03	0.12	0.37	0.87	18.98	0.35 (3.37)
Sum DEHP	NA	0.01	0.08	0.12	0.2	7.59	0.14 (2.43)

**Figure A5:**
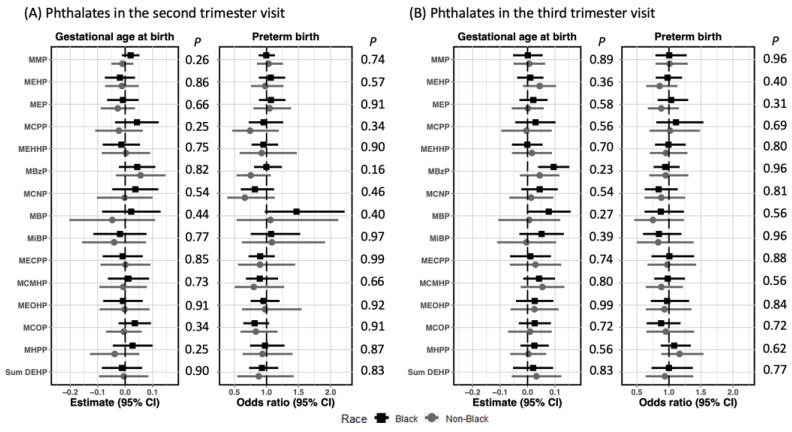
Estimated associations between prenatal phthalates and birth outcomes from the fully adjusted models in CANDLE participants with a spontaneous labor, stratified by maternal race. The interaction models were adjusted for maternal age at delivery, maternal education (high school and less vs. above high school), income adjusted for household size, marital status (Married vs. Widowed/Divorced/Separated/Never married vs. Living with partner), insurance coverage (No insurance/Medicare or Medicaid Only vs. Medicare/Medicaid + Private/Private only), SG adjusted urinary cotinine measurements in the second trimester, medical history of preterm birth (Yes vs. No), pre-pregnancy body mass index (BMI) class (Under vs. normal vs. overweight vs. obesity), parity (0 vs. 1/2 vs. >=3), and the interaction term between maternal race (Black vs. non-Black) and individual phthalates. The estimates were obtained from the models with continuous gestational age, and were interpreted as two-fold increase for each individual phthalate. The odds ratio was interpreted as two-fold increase for each individual phthalate. The p value is for the interaction term.

## Data Availability

Not applicable.

## Appendix B. Tables

**Table A1:** Baseline characteristics of CANDLE participants with complete data of phthalate measurements in either visit and birth outcome (*N* = 1125).

Variables	Total (*N* = 1125)	
	N/Mean	%/SD
Maternal age (years)	26.6	5.5
Race		
African American	694	61.7%
White	362	32.2%
Asian	9	0.8%
American Indian/Alaska Native	0	0.0%
Multiple	57	5.1%
Other	3	0.3%
Missing	0	0.0%
Education level		
<High School	126	11.2%
High School/GED	521	46.3%
Technical School	342	30.4%
College Degree	135	12.0%
Grad/Professional Degree	0	0.0%
missing	1	0.1%
Marital status		
Married	440	39.1%
Widowed/Divorced/Separated/Never married	488	43.4%
Living with partner	196	17.4%
missing	1	0.1%
Household income		
$0–$24,999	447	39.7%
$25,000–$44,999	192	17.1%
$45,000–$74,999	221	19.6%
>=$75,000	180	16.0%
missing	85	7.6%
Health insurance coverage		
No insurance	2	0.2%
Medicaid and/or Medicare only	623	55.4%
Medicaid/Medicare and Private	36	3.2%
Private Only	464	41.2%
Parity		
0	451	40.1%
1–2	532	47.3%
3–5	132	11.7%
>=6	10	0.9%
Prior preterm birth(s)		
0	1036	92.1%
1	81	7.2%
2	5	0.4%
3	3	0.3%
Pregnancy urinary cotinine (ng/mL)	64.1	316.7
Pre-pregnancy BMI (kg/m^2^)	27.7	7.7
Intimate partner violence (count of types of violence reported)		
0	311	27.6%
1	563	50.0%
2	124	11.0%
3	60	5.3%
4	19	1.7%
Missing	48	4.3%

Abbreviations: BMI, Body Mass Index; GED, Graduate Equivalency Degree.

**Table A2:** Distributions of specific gravity adjusted urinary phthalate concentrations in CANDLE by maternal race.

Metabolites	Geometric Mean (ng/mL) (SD) in the Second Trimester Visit		Geometric Mean (ng/mL) (SD) in the Third Trimester Visit	
	Black	Non-Black	Black	Non-Black
MMP	2.42 (8.04)	2.57 (7.46)	2.2 (3.32)	1.78 (3.79)
MEHP	8.8 (3.07)	6.14 (3.73)	2.73 (4.23)	1.5 (3.74)
MEP	167.26 (3.1)	92.22 (3.56)	165.02 (3.44)	82.47 (4.12)
MCPP	2.24 (2.23)	2.64 (2.48)	1.48 (2.39)	1.59 (2.27)
MEHHP	31.81 (2.42)	31.91 (2.48)	9.05 (3.18)	8.1 (2.72)
MBzP	24.18 (2.53)	17.96 (2.55)	11.85 (2.92)	7.95 (3.13)
MCNP	3.52 (2.22)	3.66 (2.18)	0.55 (2.48)	0.48 (2.53)
MBP	36.51 (1.94)	26.9 (1.81)	18.19 (2.1)	13.05 (2.05)
MiBP	14.68 (2.02)	11.02 (1.99)	8.58 (2.21)	6.19 (2.08)
MECPP	20.11 (2.33)	21.72 (2.42)	13.53 (2.5)	12.7 (2.27)
MCMHP	19.39 (2.37)	20.37 (2.46)	7.01 (2.87)	6.28 (2.91)
MEOHP	15.32 (2.35)	15.99 (2.45)	6.96 (2.68)	6.6 (2.35)
MCOP	14.67 (3.19)	18.2 (3.48)	2.51 (3.04)	2.55 (3.05)
MHPP	1.16 (2.41)	1.32 (2.42)	0.36 (3.4)	0.33 (3.34)
Sum DEHP	0.34 (2.32)	0.34 (2.43)	0.14 (2.53)	0.13 (2.28)

**Table A3:** Estimated effects of the interaction between Intimate partner violence and individual phthalates in each visit.

	Gestational Week		Preterm Birth	
	IPV*Phthalate in the 1st Visit	IPV*Phthalate in the 2nd Visit	IPV*Phthalate in the 1st Visit	IPV*Phthalate in the 2nd Visit
	Estimate (95% CI)	Estimate (95% CI)	OR (95% CI)	OR (95% CI)
MMP	0.02 (−0.01, 0.05)	−0.001 (−0.06, 0.05)	0.95 (0.87, 1.04)	1.03 (0.88, 1.19)
MEHP	−0.003 (−0.04, 0.04)	0.01 (−0.04, 0.06)	1.02 (0.88, 1.18)	1.07 (0.94, 1.22)
MEP	−0.01 (−0.06, 0.03)	0.001 (−0.04, 0.04)	1.00 (0.87, 1.15)	1.03 (0.91, 1.17)
MCPP	0.002 (−0.07, 0.07)	−0.02 (−0.09, 0.05)	1.05 (0.85, 1.3)	1.07 (0.89, 1.27)
MEHHP	−0.02 (−0.08, 0.05)	0.01 (−0.06, 0.08)	0.99 (0.79, 1.24)	1.05 (0.87, 1.26)
MBzP	−0.02 (−0.08, 0.04)	0.01 (−0.04, 0.07)	0.97 (0.81, 1.16)	1.01 (0.87, 1.17)
MCNP	0.01 (−0.07, 0.08)	−0.01 (−0.08, 0.06)	1.11 (0.89, 1.38)	1.04 (0.87, 1.25)
MBP	−0.03 (−0.12, 0.07)	0.004 (−0.08, 0.09)	0.98 (0.74, 1.29)	1.08 (0.86, 1.37)
MiBP	−0.01 (−0.08, 0.07)	0.002 (−0.08, 0.08)	0.95 (0.76, 1.2)	1.11 (0.88, 1.4)
MECPP	−0.01 (−0.07, 0.06)	0.01 (−0.07, 0.09)	1.00 (0.80, 1.25)	1.06 (0.86, 1.3)
MCMHP	−0.01 (−0.08, 0.06)	0.02 (−0.06, 0.09)	1.07 (0.86, 1.33)	0.98 (0.8, 1.21)
MEOHP	−0.02 (−0.08, 0.05)	0.01 (−0.06, 0.09)	0.98 (0.78, 1.25)	1.06 (0.86, 1.31)
MCOP	0.01 (−0.04, 0.07)	−0.01 (−0.06, 0.05)	1.06 (0.92, 1.23)	1.05 (0.90, 1.21)
MHPP	−0.03 (−0.09, 0.04)	−0.01 (−0.06, 0.04)	1.06 (0.86, 1.3)	1.06 (0.93, 1.22)
Sum DEHP	−0.01 (−0.08, 0.06)	0.01 (−0.07, 0.09)	1.02 (0.8, 1.28)	1.07 (0.86, 1.33)

The interaction models were adjusted for maternal race (Black vs. non-Black), maternal age at delivery, maternal education (high school and less vs. above high school), income adjusted for household size, marital status (Married vs. Widowed/Divorced/Separated/Never married vs. Living with partner), insurance coverage (No insurance/Medicare or Medicaid Only vs. Medicare/Medicaid + Private/Private only), SG adjusted urinary cotinine measurements in the second trimester, medical history of preterm birth (Yes vs. No), pre-pregnancy body mass index (BMI) class (Under vs. normal vs. overweight vs. obesity), parity (0 vs. 1/2 vs. >=3), and the interaction term between Intimate partner violence and individual phthalates. The estimates were obtained from the models with continuous gestational age, and were interpreted as two-fold increase for each individual phthalate. The odds ratio was interpreted as two-fold increase for each individual phthalate. Abbreviations: IPV: Intimate partner violence; OR: Odds ratio; CI: Confidence interval.

**Table A4:** Estimated effects of the interaction between Intimate partner violence and individual phthalates in each visit in CANDLE participants with a spontaneous labor.

	Gestational Week		Preterm Birth	
	IPV*Phthalate in the 1st Visit	IPV*Phthalate in the 2nd Visit	IPV*Phthalate in the 1st Visit	IPV*Phthalate in the 2nd Visit
	Estimate (95% CI)	Estimate (95% CI)	OR (95% CI)	OR (95% CI)
MMP	0.01 (−0.02, 0.04)	−0.01 (−0.06, 0.04)	0.95 (0.87, 1.04)	1.03 (0.88, 1.19)
MEHP	−0.004 (−0.04, 0.04)	0.004 (−0.04, 0.05)	1.02 (0.88, 1.18)	1.07 (0.94, 1.22)
MEP	−0.01 (−0.05, 0.04)	0.003 (−0.04, 0.04)	1.00 (0.87, 1.15)	1.03 (0.91, 1.17)
MCPP	0.01 (−0.06, 0.09)	−0.02 (−0.08, 0.04)	1.05 (0.85, 1.30)	1.07 (0.89, 1.27)
MEHHP	−0.02 (−0.09, 0.04)	0.003 (−0.06, 0.06)	0.99 (0.79, 1.24)	1.05 (0.87, 1.26)
MBzP	−0.02 (−0.08, 0.04)	0.01 (−0.04, 0.05)	0.97 (0.81, 1.16)	1.01 (0.87, 1.17)
MCNP	0.04 (−0.04, 0.12)	−0.02 (−0.08, 0.04)	1.11 (0.89, 1.38)	1.04 (0.87, 1.25)
MBP	−0.03 (−0.13, 0.06)	−0.01 (−0.08, 0.07)	0.98 (0.74, 1.29)	1.08 (0.86, 1.37)
MiBP	−0.003 (−0.08, 0.07)	0.004 (−0.07, 0.08)	0.95 (0.76, 1.20)	1.11 (0.88, 1.4)
MECPP	−0.01 (−0.08, 0.05)	−0.003 (−0.07, 0.07)	1.00 (0.8, 1.25)	1.06 (0.86, 1.3)
MCMHP	−0.003 (−0.07, 0.07)	0.01 (−0.06, 0.08)	1.07 (0.86, 1.33)	0.98 (0.8, 1.21)
MEOHP	−0.03 (−0.09, 0.04)	0.01 (−0.06, 0.07)	0.98 (0.78, 1.25)	1.06 (0.86, 1.31)
MCOP	0.03 (−0.02, 0.08)	−0.01 (−0.06, 0.04)	1.06 (0.92, 1.23)	1.05 (0.9, 1.21)
MHPP	−0.02 (−0.08, 0.05)	−0.02 (−0.06, 0.03)	1.06 (0.86, 1.30)	1.06 (0.93, 1.22)
Sum DEHP	−0.02 (−0.09, 0.05)	0.003 (−0.07, 0.07)	1.02 (0.80, 1.28)	1.07 (0.86, 1.33)

The interaction models were adjusted for maternal race (Black vs. non-Black), maternal age at delivery, maternal education (high school and less vs. above high school), income adjusted for household size, marital status (Married vs. Widowed/Divorced/Separated/Never married vs. Living with partner), insurance coverage (No insurance/Medicare or Medicaid Only vs. Medicare/Medicaid + Private/Private only), SG adjusted urinary cotinine measurements in the second trimester, medical history of preterm birth (Yes vs. No), pre-pregnancy body mass index (BMI) class (Under vs. normal vs. overweight vs. obesity), parity (0 vs. 1/2 vs. >=3), and the interaction term between Intimate partner violence and individual phthalates. The estimates were obtained from the models with continuous gestational age, and were interpreted as two-fold increase for each individual phthalate. The odds ratio was interpreted as two-fold increase for each individual phthalate. Abbreviations: IPV: Intimate partner violence; OR: Odds ratio; CI: Confidence interval.

## References

[1] Ferguson K.K., McElrath T.F., Meeker J.D. (2014). Environmental Phthalate Exposure and Preterm Birth. JAMA Pediatr..

[2] American College of Obstetricians and Gynecologists (2021). Prediction and Prevention of Spontaneous Preterm Birth: ACOG Practice Bulletin, Number 234. Obstet. Gynecol..

[3] Martin J.A., Hamilton B.E., Osterman M.J.K. (2017). Births in the United States, 2016. NCHS Data Brief..

[4] National Vital Statistics Reports, Volume 66, Number 1, January 5, 2017. 70p. https://www.cdc.gov/nchs/data/nvsr/nvsr66/nvsr66_01.pdf.

[5] Sharma D., Padmavathi I.V., Tabatabaii S.A., Farahbakhsh N. (2021). Late Preterm: A New High Risk Group in Neonatology. J. Matern. Fetal Neonatal Med..

[6] Tabet M., Jakhar S., Williams C.A., Rawat U., Hailegiorgis Y.D., Flick L.H., Chang J.J. (2017). Racial/Ethnic Differences in Correlates of Spontaneous and Medically-Indicated Late Preterm Births among Adolescents. J. Pediatr. Adolesc. Gynecol..

[7] Trilla C.C., Medina M.C., Ginovart G., Betancourt J., Armengol J.A., Calaf J. (2014). Maternal Risk Factors and Obstetric Complications in Late Preterm Prematurity. Eur. J. Obstet. Gynecol. Reprod. Biol..

[8] Staneva A., Bogossian F., Pritchard M., Wittkowski A. (2015). The Effects of Maternal Depression, Anxiety, and Perceived Stress during Pregnancy on Preterm Birth: A Systematic Review. Women Birth.

[9] Woodruff T.J., Zota A.R., Schwartz J.M. (2011). Environmental Chemicals in Pregnant Women in the United States: NHANES 2003–2004. Environ. Health Perspect..

[10] American College of Obstetricians and Gynecologists and Committee on Obstetric Practice (2021). Reducing Prenatal Exposure to Toxic Environmental Agents: ACOG Committee Opinion, Number 832. Obstet. Gynecol..

[11] Marie C., Vendittelli F., Sauvant-Rochat M.-P. (2015). Obstetrical Outcomes and Biomarkers to Assess Exposure to Phthalates: A Review. Environ. Int..

[12] Sathyanarayana S. (2008). Phthalates and Children’s Health. Curr. Probl. Pediatr. Adolesc. Health Care.

[13] Zota A.R., Shamasunder B. (2017). The Environmental Injustice of Beauty: Framing Chemical Exposures from Beauty Products as a Health Disparities Concern. Am. J. Obstet. Gynecol..

[14] Wenzel A.G., Brock J.W., Cruze L., Newman R.B., Unal E.R., Wolf B.J., Somerville S.E., Kucklick J.R. (2018). Prevalence and Predictors of Phthalate Exposure in Pregnant Women in Charleston, SC. Chemosphere.

[15] Creasy and Resnik’s Maternal-Fetal Medicine: Principles and Practice—8th Edition. https://www.elsevier.com/books/creasy-and-resniks-maternal-fetal-medicine-principles-and-practice/resnik/978-0-323-47910-3.

[16] Nishioka J., Iwahara C., Kawasaki M., Yoshizaki F., Nakayama H., Takamori K., Ogawa H., Iwabuchi K. (2012). Di-(2-Ethylhexyl) Phthalate Induces Production of Inflammatory Molecules in Human Macrophages. Inflamm. Res..

[17] Hurst C.H., Waxman D.J. (2003). Activation of PPAR and PPAR by Environmental Phthalate Monoesters. Toxicol. Sci..

[18] Ferguson K.K., McElrath T.F., Chen Y.-H., Mukherjee B., Meeker J.D. (2015). Urinary Phthalate Metabolites and Biomarkers of Oxidative Stress in Pregnant Women: A Repeated Measures Analysis. Environ. Health Perspect..

[19] Meeker J.D., Hu H., Cantonwine D.E., Lamadrid-Figueroa H., Calafat A.M., Ettinger A.S., Hernandez-Avila M., Loch-Caruso R., Téllez-Rojo M.M. (2009). Urinary Phthalate Metabolites in Relation to Preterm Birth in Mexico City. Environ. Health Perspect..

[20] Adibi J.J., Hauser R., Williams P.L., Whyatt R.M., Calafat A.M., Nelson H., Herrick R., Swan S.H. (2009). Maternal Urinary Metabolites of Di-(2-Ethylhexyl) Phthalate in Relation to the Timing of Labor in a US Multicenter Pregnancy Cohort Study. Am. J. Epidemiol..

[21] Wolff M.S., Engel S.M., Berkowitz G.S., Ye X., Silva M.J., Zhu C., Wetmur J., Calafat A.M. (2008). Prenatal Phenol and Phthalate Exposures and Birth Outcomes. Environ. Health Perspect..

[22] Burdorf A., Brand T., Jaddoe V.W., Hofman A., Mackenbach J.P., Steegers E.A.P. (2011). The Effects of Work-Related Maternal Risk Factors on Time to Pregnancy, Preterm Birth and Birth Weight: The Generation R Study. Occup. Environ. Med..

[23] Huang P.-C., Kuo P.-L., Chou Y.-Y., Lin S.-J., Lee C.-C. (2009). Association between Prenatal Exposure to Phthalates and the Health of Newborns. Environ. Int..

[24] Suzuki Y., Niwa M., Yoshinaga J., Mizumoto Y., Serizawa S., Shiraishi H. (2010). Prenatal Exposure to Phthalate Esters and PAHs and Birth Outcomes. Environ. Int..

[25] Welch B.M., Keil A.P., Buckley J.P., Calafat A.M., Christenbury K.E., Engel S.M., O’Brien K.M., Rosen E.M., James-Todd T., Zota A.R. (2022). Associations Between Prenatal Urinary Biomarkers of Phthalate Exposure and Preterm Birth: A Pooled Study of 16 US Cohorts. JAMA Pediatr..

[26] Coussons-Read M.E., Lobel M., Carey J.C., Kreither M.O., D’Anna K., Argys L., Ross R.G., Brandt C., Cole S. (2012). The Occurrence of Preterm Delivery Is Linked to Pregnancy-Specific Distress and Elevated Inflammatory Markers across Gestation. Brain. Behav. Immun..

[27] Hedegaard M., Henriksen T.B., Sabroe S., Secher N.J. (1993). Psychological Distress in Pregnancy and Preterm Delivery. BMJ.

[28] Rondó P.H.C., Ferreira R.F., Nogueira F., Ribeiro M.C.N., Lobert H., Artes R. (2003). Maternal Psychological Stress and Distress as Predictors of Low Birth Weight, Prematurity and Intrauterine Growth Retardation. Eur. J. Clin. Nutr..

[29] Donovan B.M., Spracklen C.N., Schweizer M.L., Ryckman K.K., Saftlas A.F. (2016). Intimate Partner Violence during Pregnancy and the Risk for Adverse Infant Outcomes: A Systematic Review and Meta-Analysis. BJOG Int. J. Obstet. Gynaecol..

[30] Hill A., Pallitto C., McCleary-Sills J., Garcia-Moreno C. (2016). A Systematic Review and Meta-Analysis of Intimate Partner Violence during Pregnancy and Selected Birth Outcomes. Int. J. Gynecol. Obstet..

[31] do Prado C.H., Grassi-Oliveira R., Wieck A., Zaparte A., Filho L.D., da Silva Morrone M., Moreira J.C., Bauer M.E. (2016). The Impact of Childhood Maltreatment on Redox State: Relationship with Oxidative Damage and Antioxidant Defenses in Adolescents with No Psychiatric Disorder. Neurosci. Lett..

[32] Rosen E.M., van’t Erve T.J., Boss J., Sathyanarayana S., Barrett E.S., Nguyen R.H.N., Bush N.R., Milne G.L., McElrath T.F., Swan S.H. (2019). Urinary Oxidative Stress Biomarkers and Accelerated Time to Spontaneous Delivery. Free Radic. Biol. Med..

[33] Guendelman S., Lang Kosa J., Pearl M., Graham S., Kharrazi M. (2008). Exploring the Relationship of Second-Trimester Corticotropin Releasing Hormone, Chronic Stress and Preterm Delivery. J. Matern. Fetal Neonatal Med..

[34] Sontag-Padilla L.M., Burns R.M., Shih R.A., Griffin B.A., Martin L.T., Chandra A., Tylavsky F. (2015). The Urban Child Institute CANDLE Study: Methodological Overview and Baseline Sample Description.

[35] LeWinn K.Z., Karr C.J., Hazlehurst M., Carroll K., Loftus C., Nguyen R., Barrett E., Swan S.H., Szpiro A.A., Paquette A. (2022). Cohort Profile: The ECHO Prenatal and Early Childhood Pathways to Health Consortium (ECHO-PATHWAYS). BMJ Open.

[36] Suman V., Luther E.E. (2021). Preterm Labor.

[37] Straus M.A., Hamby S.L., Boney-McCoy S., Sugarman D.B. (1996). The Revised Conflict Tactics Scales (CTS2): Development and Preliminary Psychometric Data. J. Fam. Issues.

[38] Rocha B.A., Asimakopoulos A.G., Barbosa F., Kannan K. (2017). Urinary Concentrations of 25 Phthalate Metabolites in Brazilian Children and Their Association with Oxidative DNA Damage. Sci. Total Environ..

[39] Davis R.A., Stiles M.F., de Bethizy J.D., Reynolds J.H. (1991). Dietary Nicotine: A Source of Urinary Cotinine. Food Chem. Toxicol..

[40] Azur M.J., Stuart E.A., Frangakis C., Leaf P.J. (2011). Multiple Imputation by Chained Equations: What Is It and How Does It Work?. Int. J. Methods Psychiatr. Res..

[41] Burniaux J.-M., Dang T.-T., Fore D., Förster M.F., Mira D’Ercole M., Oxley H. (1998). Income Distribution and Poverty in Selected OECD Countries.

[42] Hastie T., Tibshirani R. (1995). Generalized Additive Models for Medical Research. Stat. Methods Med. Res..

[43] NHANES 2009–2010: Phthalates—Urine Data Documentation, Codebook, and Frequencies. https://wwwn.cdc.gov/nchs/nhanes/2009-2010/PHTHTE_F.htm.

[44] Ferguson K.K., Rosen E.M., Rosario Z., Feric Z., Calafat A.M., McElrath T.F., Vega C.V., Cordero J.F., Alshawabkeh A., Meeker J.D. (2019). Environmental Phthalate Exposure and Preterm Birth in the PROTECT Birth Cohort. Environ. Int..

[45] Ferguson K.K., Rosen E.M., Barrett E.S., Nguyen R.H.N., Bush N., McElrath T.F., Swan S.H., Sathyanarayana S. (2019). Joint Impact of Phthalate Exposure and Stressful Life Events in Pregnancy on Preterm Birth. Environ. Int..

[46] McCarthy F.P., Delany A.C., Kenny L.C., Walsh S.K. (2013). PPAR-γ—A Possible Drug Target for Complicated Pregnancies. Br. J. Pharmacol..

[47] Buckley J.P., Palmieri R.T., Matuszewski J.M., Herring A.H., Baird D.D., Hartmann K.E., Hoppin J.A. (2012). Consumer Product Exposures Associated with Urinary Phthalate Levels in Pregnant Women. J. Expo. Sci. Environ. Epidemiol..

[48] Sathyanarayana S., Focareta J., Dailey T., Buchanan S. (2012). Environmental Exposures: How to Counsel Preconception and Prenatal Patients in the Clinical Setting. Am. J. Obstet. Gynecol..

[49] Bellinger D.C. (2005). Teratogen Update: Lead and Pregnancy. Birt. Defects Res. A Clin. Mol. Teratol..

[50] Rahman A., Kumarathasan P., Gomes J. (2016). Infant and Mother Related Outcomes from Exposure to Metals with Endocrine Disrupting Properties during Pregnancy. Sci. Total Environ..

[51] Xue F., Holzman C., Rahbar M.H., Trosko K., Fischer L. (2007). Maternal Fish Consumption, Mercury Levels, and Risk of Preterm Delivery. Environ. Health Perspect..

[52] Sathyanarayana S., Butts S., Wang C., Barrett E., Nguyen R., Schwartz S.M., Haaland W., Swan S.H. (2017). Early Prenatal Phthalate Exposure, Sex Steroid Hormones, and Birth Outcomes. J. Clin. Endocrinol. Metab..

[53] Cantonwine D.E., Meeker J.D., Ferguson K.K., Mukherjee B., Hauser R., McElrath T.F. (2016). Urinary Concentrations of Bisphenol A and Phthalate Metabolites Measured during Pregnancy and Risk of Preeclampsia. Environ. Health Perspect..

[54] Werner E.F., Braun J.M., Yolton K., Khoury J.C., Lanphear B.P. (2015). The Association between Maternal Urinary Phthalate Concentrations and Blood Pressure in Pregnancy: The HOME Study. Environ. Health.

[55] Patelarou E., Kelly F.J. (2014). Indoor Exposure and Adverse Birth Outcomes Related to Fetal Growth, Miscarriage and Prematurity—A Systematic Review. Int. J. Environ. Res. Public. Health.

